# Neurenteric Cyst of the Area Postrema

**DOI:** 10.1155/2014/718415

**Published:** 2014-09-16

**Authors:** Claire M. Miller, Bonnie H. Wang, Seong-Jin Moon, Eric Chen, Huan Wang

**Affiliations:** ^1^Neuroscience Program, University of Illinois College of Medicine at Urbana-Champaign, Urbana, IL 61801, USA; ^2^Department of Internal Medicine, University of Illinois College of Medicine at Urbana-Champaign, Urbana, IL 61801, USA; ^3^University of Illinois College of Medicine at Urbana-Champaign, Urbana, IL 61801, USA; ^4^Division of Biology and Medicine, Brown University, Providence, RI 02912, USA; ^5^Department of Neurosurgery, University of Illinois College of Medicine at Urbana-Champaign, Carle Foundation Hospital, 602 West University Avenue, Urbana, IL 61801, USA

## Abstract

Neurenteric cysts are CNS lesions most frequently occurring in the spinal cord. Intracranial neurenteric cysts are rarer, typically presenting with headache, mass effect, or location-specific symptoms. The area postrema is known as the emetic center of the brain; lesions can cause nausea and vomiting. Our case, featuring a neurenteric cyst of the area postrema, illustrates the importance of considering a neurological etiology for nonspecific symptoms that otherwise elude explanation. Our patient presented with acute decompensated hydrocephalus upon exploratory abdominal laparoscopy for unresolving abdominal pain. The patient had an eight-month history of unexplained intermittent nausea, vomiting, and abdominal pain. These bouts increased in frequency during the weeks before acute presentation, prompting exploratory abdominal laparoscopy. The acute decompensation was managed by ventriculostomy, and cranial MRI revealed a cystic mass by the floor of the fourth ventricle. After the patient stabilized and returned to neurological baseline, suboccipital craniectomy and resection were performed. The mass was histologically identified as a neurenteric cyst. The patient was free from neurological complaints at one-year follow-up, indicating that the successful resection of the area postrema-associated neurenteric cyst resolved her previous symptoms. Thus, some intracranial lesions can masquerade as nonspecific symptoms, presenting a challenge to accurate diagnosis.

## 1. Introduction

Neurenteric cysts (NCs) are rare congenital abnormalities occurring within the central nervous system. They most frequently present as lesions of the ventral spine [1, 2] and are histopathologically characterized by features of intestinal or respiratory epithelium [3]. Only a small number of NCs occur intracranially, representing about 0.01–0.35% of all CNS lesions [4, 5]. NCs are predominantly reported by case study [4–7], and the reports feature nonspecific symptomology that is commonly attributed to mass effect associated with lesion location [6]. The most frequently presenting symptoms include headache, numbness, weakness, nausea, specific nerve defects, and, more rarely, aseptic meningitis [8]. Noted onset of symptoms ranges from one week to 10 years prior to diagnosis; many histories tend to include intermittent or waxing and waning features [5]. Thus, the full range of potential clinical and pathological features must be appreciated to aid in future awareness and diagnosis of these rare cysts. Furthermore, because patient presentation depends more on location and mass effect than on an NC tissue-specific attribute, understanding the nonspecific presentation typical of these cysts will contribute to differential diagnosis inclusive of any benign neurological cyst with mass effect.

We present a case of NC adherent to the area postrema (AP), successfully treated surgically. The AP is an emesis-inducing center in the medulla, located at the caudate floor of the fourth ventricle. AP tissue is densely vascularized and lacks tight junctions, enabling the detection of toxins in both blood and cerebrospinal fluid (CSF) [9–12]. Although well characterized from anatomical and physiological perspectives, there is a relative dearth of literature pertaining to mass lesions of the AP in the clinical setting. Our patient presented episodically with severe nausea, vomiting, and abdominal pain, yet without any associated neurological symptoms initially. The nonspecific nature of the presenting symptoms focused the differential diagnosis away from the nervous system. Although we could not explicitly ascribe the presenting symptoms in this case study to mass versus local chemical effect on the AP, our case highlights the importance of considering a neurological origin for nonspecific symptoms with an elusive etiology.

## 2. Case Report

### 2.1. History

A 34-year-old Caucasian female presented with an eight-month history of frequent emergency department (ED) visits, related to episodically severe abdominal pain with nausea and vomiting. Within several weeks prior to presentation, she presented at the ED four times for migrating abdominal pain with associated nausea and vomiting. During that time, she was treated for gastroenteritis and irritable bowel syndrome but continued experiencing abdominal pain and nausea. With each ED visit, she underwent similar clinical and imaging evaluations that focused on the chief complaint of nausea, vomiting, and abdominal pain (urine tests, ultrasound, CT of abdomen and pelvis, etc.).

Several ovarian cysts, thought to be physiologic, were identified via abdominal scan. Finally, exploratory laparoscopy was performed. The following day, she exhibited progressive neurological deterioration. Cranial CT demonstrated hydrocephalus. In an obtunded state, she was emergently transferred to neurosurgery service.

### 2.2. Examination

Upon physical examination, she was unresponsive to verbal cue. She was able to moan and to move all four extremities. Her pupils were large and sluggishly reactive.

### 2.3. Operation

**Figure 1:**
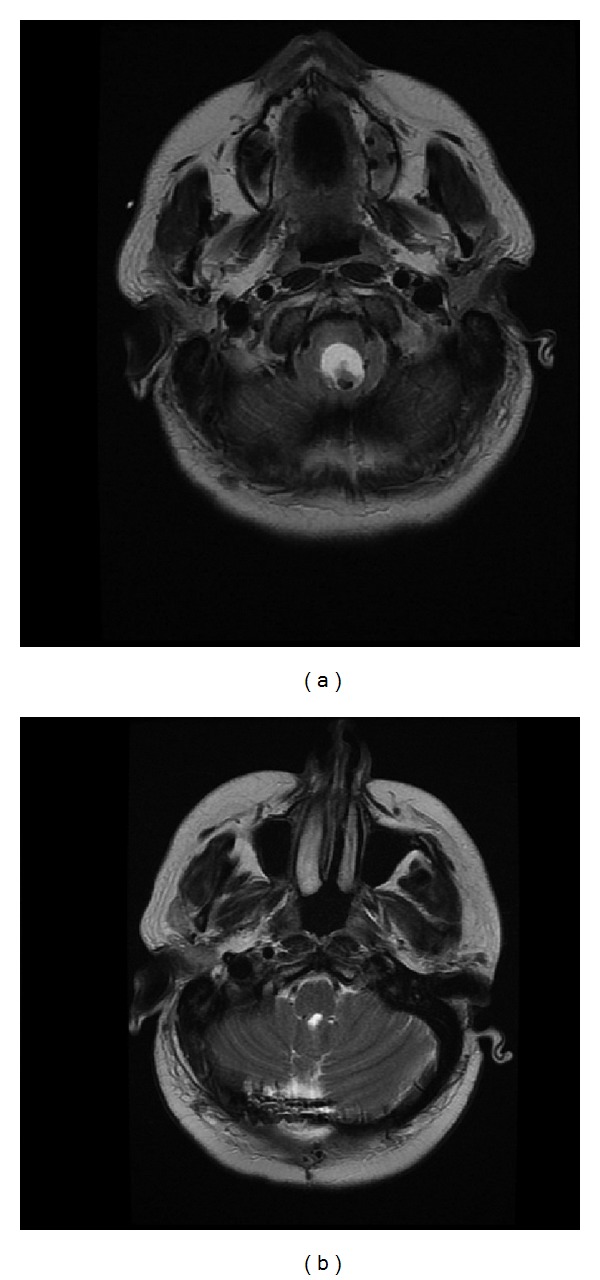
(a) Preoperative T_2_-weighted MRI with axial view depicting an ellipsoid hyperintense cystic mass, hydrocephalus, and brainstem compression. (b) Postoperative T_2_-weighted MRI with axial view depicting successful resection of mass.

**Figure 2:**
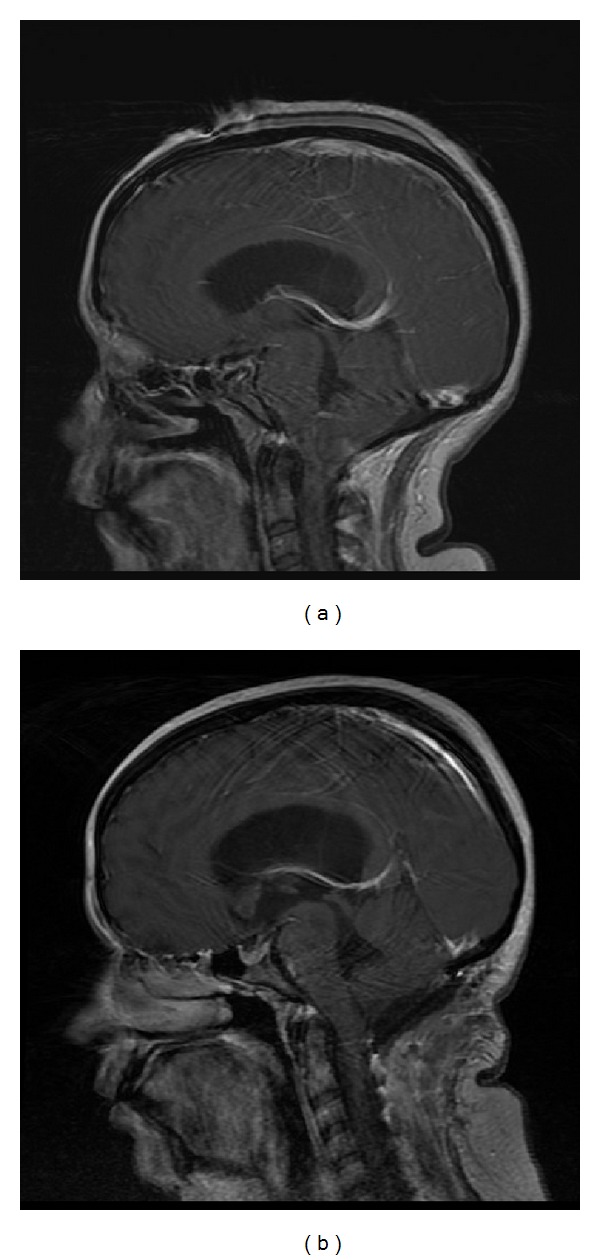
(a) Preoperative T_1_-weighted MRI with sagittal view. (b) Postoperative T_1_-weighted MRI with sagittal view.

She underwent emergent ventriculostomy and returned to her normal neurological baseline within 24 hours. Cranial MRI evaluation demonstrated a cystic mass near the caudal region of the fourth ventricle, resulting in obstructive hydrocephalus and brainstem compression (Figures 1(a) and 2(a)).

She subsequently underwent suboccipital craniectomy for gross total tumor resection. Intraoperatively, after the arachnoid overlying the cistern magna was opened and the cystic mass was punctured and well decompressed, the cyst wall was dissected free from surrounding brain, including the floor of the fourth ventricle and cerebellum. The cyst wall was noted to be particularly adherent to the AP.

### 2.4. Pathological Findings

Microscopic examination of the dissected tissue revealed a cystic mass lined by mucous columnar cells, surrounding proteinaceous material, calcifications, vascular ectasia, and reactive changes with embedded small glandular structures. The diagnosis of NC was confirmed (please refer to Section 4).

### 2.5. Postoperative Course

Her symptoms of nausea, vomiting, and abdominal pain were all resolved on postoperative day 1. She was discharged home in 5 days uneventfully. She made an excellent recovery without a clinical need for a ventriculoperitoneal shunt. At her most recent one-year follow-up visit, she reported no neurological concerns. Follow-up cranial MRI confirmed the complete removal of the cystic mass (Figures 1(b) and 2(b)).

## 3. Discussion

NCs are benign and rare masses of the CNS [4, 5]. They most frequently occur in the spinal cord, followed by infratentorial and then supratentorial locations [5, 7, 12]. Histologically, they have features of gastrointestinal or respiratory epithelium, which has led to a variety of names, including intestinome and enterogenic or bronchogenic cyst, over the years [4]. NCs typically stain positive for cytokeratin, EMA, and CEA. CA19-9 is also sometimes used; however, there is no specific or definitive NC-associated marker [13, 14]. Baker and Bernat classified spinal cord NCs into three histological types: Type A cysts feature columnar or cuboidal epithelium with or without ciliation; Type B cysts also include mucinous or serous glands and fluid production; and Type C cysts also include ependymal or glial tissue [3]. No such explicit classification schema is available for intracranial NCs, though they share the histological features of spinal cord NCs. Most frequently, the diagnosis of NC is definitively established based on histopathological characteristics observed during or after resection of the lesion.

Although diagnosis of NC must be based on histology, radiological characteristics, which are typically known prior to cyst resection and histological assessment, are also important. NCs can be difficult to differentiate from other cysts, such as arachnoid cysts, epidermoid cysts, and other endodermal cysts such as Rathke cleft cysts or colloid cysts [15]. While arachnoid cysts are often treated without surgical intervention, total surgical resection is strongly recommended for NCs. Radiologically, the presence of a nonenhancing, round lobule that is isointense or marginally hyperintense relative to CSF on T_1_-weighted MRI scans and vastly hyperintense on T_2_-weighted MRI scans suggests the presence of an NC [5, 13, 14, 16]. The radiological characteristics of NCs are likely due to the high proteinaceous content of the cysts, as well as fluid mobility within the cyst [15]. NCs are also hyperintense on FLAIR imaging. Location and characteristics of the cyst (such as appearance and growth pattern) can offer clues to an effective differential diagnosis.

NCs present a diagnostic challenge as they occur rarely and symptomology depends on location rather than an intrinsic feature of the cyst. A rare tumor with common and/or nonspecific presentation can be difficult to diagnose, and indeed there is a documented case of spinal NC presenting as demyelinating disease [17]. As of 2012, there exist only approximately 100 published records of intracranial NCs for the past 50+ years [4, 5, 7, 18]. The majority of these cysts are infratentorial; anterior fossa cysts represent less than one-third of all published cases [7]. The total number of NCs may be underdiagnosed, as the lesions themselves are benign and are typically only discovered because of symptoms secondary to mass size. In the future, incidental diagnosis of the cysts may increase as more cranial scans are routinely performed.

In the described case, the nonspecific nature of the presenting symptoms focused the differential diagnosis away from the nervous system. Because our patient initially had no neurological complaints or symptoms, she did not undergo any cranial imaging evaluation until she presented in an obtunded state from hydrocephalus. Within several weeks prior to the acute neurological deterioration, she presented at the ED four times for migrating abdominal pain with associated nausea and vomiting. Such common gastrointestinal (GI) symptoms correctly prompted diagnostic attention towards the abdomen. Although an extensive diagnostic workup was repeatedly performed and ovarian cysts were incidentally identified, an etiology for the patient's symptoms was never determined. She was treated for gastroenteritis and irritable bowel syndrome but continued experiencing abdominal pain and nausea. Such symptoms promptly resolved after surgical resection of the fourth ventricular NC. In this case, it is difficult to explicitly ascribe such presenting symptoms to mass versus local chemical effect on the AP. Notably, other authors have reported that a lesion of the AP can create nausea, vomiting, and appetite change [12].

The AP is a sensory circumventricular organ associated with the fourth ventricle, first associated with nausea and vomiting in the 1940 s/50 s [11]. Compared to other brain regions, the AP is highly vascularized. Those vessels are characterized by a leaky fenestrated epithelium and blood-filled Virchow-Robin spaces, enabling the neurons of the region to monitor the chemical composition of the peripheral bloodstream [11, 12]. Further, the AP has both afferent and efferent projections to other brainstem nuclei, most significantly the nucleus of the solitary tract and parabrachial nuclei, suggesting that it not only may be a sensory organ but also may play a role in integrating brainstem autonomic regulation and gustatory sensation [12].

Although the AP is well associated with nausea and vomiting [11, 12], and surgical lesions to the region can abate intractable vomiting [19], literature pertaining to mass lesions of the AP in the clinical setting is scarce. Takahashi et al. describe a 63-year-old male with a malignant melanoma metastasis to the right lateral margin of the pons and cerebellar peduncle, resulting in projectile vomiting that resolved four weeks after beginning corticosteroid and radiation therapy [20]. There was no evidence of hydrocephalus upon autopsy, implying that the emesis was due primarily to the location of the metastasis rather than a mass effect. Teufack et al. describe a 12-year-old male with a history of headache who presented with several months of vomiting without nausea [21]. There was no evidence of hydrocephalus although a Chiari I malformation had been previously identified. Symptoms resolved upon discovery of and operation on the left posterior inferior cerebellar artery, which was compressing the left aspect of the AP. These cases illustrate that mass lesions of the AP can be a primary cause of nausea and vomiting, rather than a secondary cause via hydrocephalus.

The AP is also clinically associated with neuromyelitis optica (NMO). The association illustrates that altered cell function can also induce nausea and vomiting. In NMO, autoantibodies against aquaporin 4, a water channel thought to be an important component of the astrocytic foot processes of the vascular epithelium of the AP, instigate an inflammatory process resulting in damage to the other cells in the area and demyelination [22, 23]. If the AP is the first significant neurological target of a person with NMO, then the initial presentation of NMO can be intractable vomiting. Also, when any case of NMO presents with symptoms of nausea and vomiting, there is likely to be a lesion of the AP [24]. Similarly to the presented case of AP-associated NC, because of the GI-associated nature of the symptomology, neurological assessment is frequently not part of the initial workup or differential diagnosis [25]. For patients known to have NMO, the onset of nausea may be a predictor of acute exacerbations [14]. Thus, the AP produces nausea and vomiting not only as a result of chemical stimulus [19], but also as a consequence of change in neural function.

## 4. Pathology

**Figure 3:**
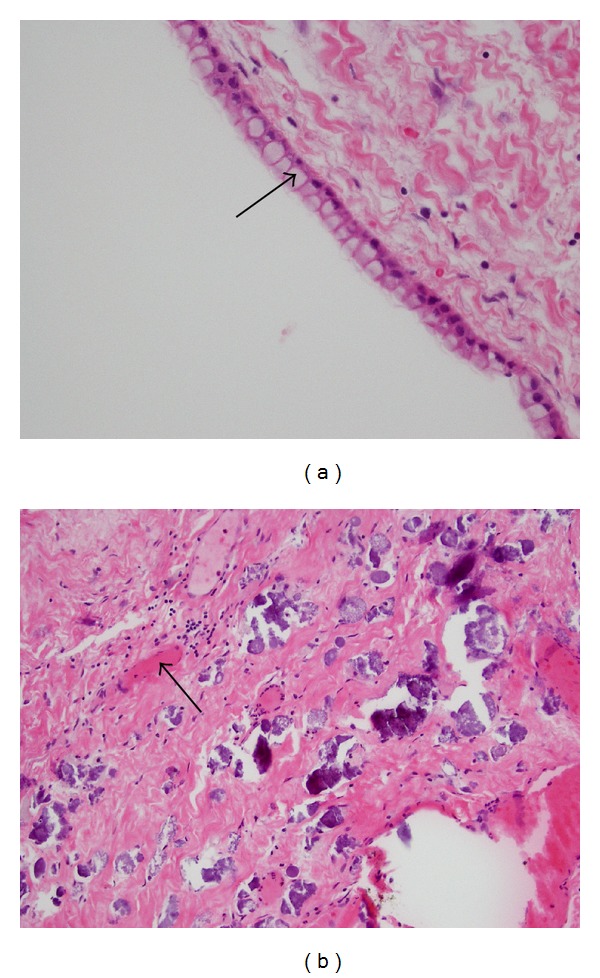
Light microscopy of the hematoxylin & eosin (H&E) stained tissue sections. (a) Histopathology of the enterogenous cyst showing simple columnar epithelium that is rich in goblet cells; original magnification ×400. (b) Granulation tissue with fibroblastic and capillary proliferation; original magnification ×100.

Hematoxylin and eosin** (**H&E) stained sections from the brain lesion revealed that cystic areas were predominantly lined by simple columnar epithelium with nonciliated cells, while cuboidal epithelium was uncommon. Goblet cells were evident throughout the epithelium (Figure 3(a)). The subepithelial fibroconnective tissue showed granulation characterized by fibroblastic and capillary proliferation and mononuclear inflammatory cell infiltrates (Figure 3(b)). Indications of dystrophic calcification and colloid-like material were also observed in the histopathology.

This case of enterogenous cyst is one of the simplest forms based on its epithelial composition of columnar and goblet cells supported by a layer of connective tissue. More complex types share a similar epithelial composition but share features of the gastrointestinal and respiratory tract [26]. Enterogenous cysts can vary in cilia abundance, inflammation, and morphology. Similar to the case published by Cheng et al., this enterogenous cyst contained debris that enclosed proteinaceous material. However, our case differs in that inflammation and dysplasia are both evident when examining the H&E stains [27]. Enterogenous cysts are readily identified based on other features such as epithelial pattern and tissue composition.

The basement membrane seen in our case suggests an endodermal origin and supports the diagnosis of enterogenous cyst [27]. The fibroblastic and capillary proliferation is also consistent with our diagnosis, given indicators of rapid cellular proliferation in previous case reports; the Ki67 proliferation fraction was over 80% in a case published by Preece et al. [16]. In our case, the histologic pattern of a single layer of ciliated columnar epithelium with goblet cells and a basement membrane is similar to the pathology of previously published cases of enterogenous cyst [27, 28]. The characteristics of our H&E stains conform to the less common of the two major histologic patterns described by Preece et al. This pattern is defined by simple, nonciliated epithelium that is rich in mucin-producing cells [28].

Immunohistochemical stains revealed the lining to be positively stained with the epithelial markers pankeratin, cytokeratin-7 (CK7), cytokeratin-20 (CK20), and epithelial membrane antigen (EMA) [26]. The positive stains for these epithelial markers confirm the staining results of previous cases [26, 27, 29, 30]. The positive stains for CK7 and CK20 suggest a foregut origin [31]. Minute foci showed positive areas for the glial marker GFAP (glial fibrillary acidic protein), which indicates reactive gliotic tissue. This is unusual because ectodermal markers such as GFAP, a subunit of astrocyte filaments and a major component in astrocyte fibers, are widely acknowledged to be unreactive in endodermal cysts [27, 29, 32]. This case of enterogenous cyst can be classified as type A according to the Wilkins-Odom classification [32], due to the simple columnar epithelium on a basal membrane without cilia and its resemblance to gastrointestinal epithelium. However, the glial elements are features of a type C cyst [16].

## 5. Conclusion

We describe an uncommon presentation of an already rare central nervous system cyst. Our patient presented with unexplained and intermittent nausea, vomiting, and abdominal pain and was found to have a NC attached to the AP, or emetic center of the brain. The AP has few associated published case studies; the presentation of our patient may be relevant to the understanding of lesions of the AP in future patients. Our case also serves as a reminder to consider a neurological etiology for common and nonspecific symptoms that lack an alternative explanation. One of the medical challenges illustrated by this case is the need to define when, in the context of nonspecific symptoms such as nausea and vomiting, a neurological etiology should be considered.

## Abbreviations

AP: Area postrema
CNS: Central nervous system
ED: Emergency department
GI: Gastrointestinal
NC: Neurenteric cyst
NMO: Neuromyelitis optica.
